# Robotic lava tube mapping and multimodal data collection using quadruped and LiDAR

**DOI:** 10.3389/frobt.2026.1855443

**Published:** 2026-07-27

**Authors:** A. J. Hidding, H. H. Bier, L. Peternel, A. J. Becoy, F. A. P. Romio, G. Calabrese

**Affiliations:** 1 Robotic Building Lab, Architecture Department, TU Delft, Delft, Netherlands; 2 Department of Cognitive Robotics, Cognitive Robotics, TU Delft, Delft, Netherlands; 3 Architecture Department, Sealine Research Centre, University of Ferrara, Ferrara, Italy; 4 Department of Engineering and Geology, IRSPS International Research School of Planetary Sciences (IRSPS), Pescara, Italy

**Keywords:** extraterrestrial habitat, lava tube, mars, quadruped, robotic mapping

## Abstract

As part of TU Delft Rhizome 2.0 and Moonshot projects, focusing on the development of extraterrestrial habitats in lava tubes, the robotic mapping of an analogue lava tube in Sicily has been studied with the future goal of assessing its suitability for building construction. The main objective of the research was to survey a lava tube and acquire a novel dataset for future research, while also analyzing the collected data to evaluate possible future lava tube exploration scenarios for the Moon and Mars. This paper presents the robotic mapping conducted in Grotta di Monte Intraleo, detailing the implementation and outlining challenges and insights, leading to the new dataset. Various mapping and data collection methods, including robotic mapping using LiDAR, was explored. The overall strategy relies on multi-technique scanning to enhance redundancy and ensure comprehensive data acquisition, that is providing a robust foundation for future robotic exploration and habitat planning.

## 1 Introduction

The European Space Agency (ESA) has set the goal of sending humans to Mars by 2040 (European Space Agency, 2023). A critical challenge in achieving this objective lies in ensuring the safety of astronauts by protecting them from radiation exposure, dramatic temperature changes, sandstorms, and micro-meteoroid impacts. Orbital observations have provided strong evidence for the existence of candidate lava tubes on Mars and the Moon, as no direct proof has been provided (Sauro et al., 2020). These could offer natural protection from the harsh Martian environment and hold potential as sites for building human habitats; hence, identifying and evaluating suitable lava tubes is a critical step in preparing for long-term human presence on Mars or the Moon.

The presented robotic mapping builds on the Rhizome 2.0 and TU Delft Moonshot projects. It contributes by analyzing the context and local conditions for a habitat + constructed from 3D-printed, prefabricated interlocking building components using *In-Situ* Resource Utilization (ISRU) and robotic assembly (Bier et al., 2025). The assembly is implemented with autonomous rovers that transport and assemble the components in the designated construction site within the lava tube. Since not all lava tubes are suitable for habitation due to size limitations, accessibility constraints, and structural integrity, scanning is one of the first tasks to implement *in situ*.

At present, limited data is available regarding extraterrestrial lava tubes. The existing data mainly consists of limited skylight observations, obtained through the High Resolution Imaging Science Experiment (HiRISE) and Context Camera (CTX) aboard the Mars Reconnaissance Orbiter (MRO), that are currently available in the Mars Global Cave Candidate Catalogue ( ${\text{MGC}}^{3}$ ) by the USGS scientist Cushing (2016). In recent years, using Light Detection and Ranging (LiDAR) technology in terrestrial lava tube surveying (Bell E. et al., 2022; Lemaire et al., 2024; Montañez Muñoz et al., 2025; Santagata et al., 2018; Santagata et al., 2023; Yang et al., 2024; Koch et al., 2024; Zhang et al., 2025), lava tube point clouds have been increasingly generated to extract data, which, in some cases, have been used to perform geotechnical analyses, estimating the stability of terrestrial lava tubes to extract insights regarding possible extraterrestrial conditions (Chwała et al., 2025). The analogues-based approach has allowed a significant advancement in planetary lava tube stability analyses, which, before, relied on idealized or synthetically generated internal lava tube morphologies (Blair et al., 2017; Chwała et al., 2024; Chwała et al., 2025; Theinat et al., 2020).

Even though there are differences between extraterrestrial and terrestrial lava tunnels, the latter currently provide the closest analogue environments available for investigation, giving insights into their extraterrestrial counterparts (Sauro et al., 2020). Extraterrestrial lava tubes would be significantly bigger, with internal diameters that could reach up to 400 m on Mars and 900–1000 m for the Moon–mainly due to lower gravity, different rheology of the lava, and higher effusion rates (Blair et al., 2017; Bunnell and Liebman, 1991; Carrer et al., 2024; Keszthelyi, 1995; Sauro et al., 2020). Exploration is a hazardous and unpredictable task; thus, initially, it is safer to send in the robot to inspect.

The primary objective of the mission is to acquire representative, high-quality LiDAR and photogrammetry data of the horizontal surfaces of a terrestrial lava tube analog, enabling both terrain assessment for rover navigation and the identification of suitable locations for habitat construction. The paper presents the research background and state of the art, followed by a description of the main contributions. Next, the methods are detailed, covering the robot hardware and Robot Operating System (ROS) 2 interface, terrain mapping and ambient sensing, robot path-planning and decision-making, backup data, and floor analysis. The results of the LiDAR scanning and the resulting dataset, including terrain analysis, are followed by a discussion on the findings, limitations, and directions for future work, and a conclusion.

## 2 Background

The Grotta di Monte Intraleo, located in the territory of Adrano on the southwestern flank of Mt. Etna, is a lava tube that may have formed in the lava flows of 1595 AD, possibly due to flow inflation and tube coalescence processes over a significant time span (Calvari and Pinkerton, 1998; Centro Speleologico Etneo, 1999). Access to the tube is possible through a collapse that is forming an entrance, measuring approximately 27 × 15 m, with a maximum depth of 8 m.

The entrance ramp consists of large, stable lava blocks left in place after the collapse of a once-existing upper level. This is evident from the vertical marks ‘striae’ visible on the walls, which were possibly still plastic and not solidified when the event occurred (Calvari and Pinkerton, 1998).

To start the mapping process, the robot was manually transported down the lava tube entrance to the start of the intact section of the tube on the south-facing wall due to the rough entrance terrain. At this point, the lava tube features three distinct levels: one upper level, inaccessible without climbing gear, and deemed irrelevant to this mission. The other two levels are accessible from the bottom of the collapse. The lower level was excluded from exploration because it contains steep jagged terrain, unsuitable for robotic navigation. The exploration, therefore, focused on the middle level, which starts off as a primary channel, extending 32 m in length and branching into three smaller passages. In this channel, diverse cross-sections exist with varying internal morphologies, and challenging terrains. On Etna, lava tubes predominantly form within ‘a’a lava flow fields, characterized by high-viscosity lava flows that form rough terrains with partly loose, irregular fragments (Calvari and Pinkerton, 1998; 1999; Halliday, 2004). Consequently, the interior of the tube, and in particular areas not completely drained during the final stages of activity, often presents rough and jagged floors with a significant presence of loose rocks, posing substantial challenges for robotic navigation.

**FIGURE 1:**
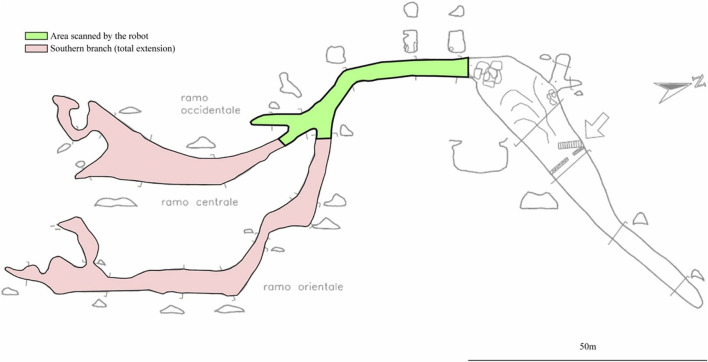
Existing survey map of Grotta di Monte Intraleo, realised with traditional speleological survey methods by Fanciulli et al. in Centro Speleologico Etneo, pp. (1999, pp. 234–236), with overlayed the surveyed area of the main southern and upper branch of the lava tube. Reproduced with permission from Centro Speleologico Etneo (1999).

The last and most up-to-date and publicly available survey map for the Grotta di Monte Intraleo lava tube was made in 1999 and provides sketched out plans and sections based on on-site measurements (Figure 1) (Fanciulli et al., 1999).

### 2.1 State-of-the-art

Various approaches to mapping lava tubes exist that can work at a distance, with some approaches relying on instruments. For example, Miyamoto et al. (2005) proposed the use of ground penetrating radar, while Bell J. F. et al. (2022) focused on detecting magnetic anomalies. Nevertheless, these methods cannot support detailed on-site mapping. An alternative approach is to rely on robotic systems that can enter lava tunnels and scan the environment up close (Walther et al., 2024). Navigation inside lava tubes introduces challenges due to low visibility, narrow passages, and various obstacles. To address these challenges, approaches such as caged drones have been developed (European Space Agency, 2017), protecting against collisions by shielding the propellers. However, their applicability on Mars is limited by the planet’s low atmospheric density, while they are not viable on the Moon due to the absence of a meaningful atmosphere. Hence, using ground robots relying on wheeled or legged systems provides a more robust solution, assuming that they are able to navigate the terrain.

In a numerical study by Olsen and Alexis (2023), the researchers used a legged robot that can jump to deal with the rough terrain. Another study used a platoon of wheeled robots and tested the system not only numerically but also experimentally in a lava tube in the Mojave Desert. Nie et al. (2024) proposed the use of a climbing ‘spider-like’ robot to enhance exploration capabilities. In recent work, Morrell et al. (2024) used two NeBula Spot quadruped robots to perform an exploration of a cave in California and a limestone mine in Kentucky that contain relatively smooth terrain features with limited exposure to rugged and three-dimensional terrains. The use of multiple robots has been identified as improving the efficiency of exploration.

### 2.2 Contribution

The main contribution of the presented lava tube mapping is an open-access dataset, containing information on the on-site exploration and mapping of the challenging terrain of Grotta di Monte Intraleo. Despite the clear advantages that LiDAR data offers scientists for conducting precise evaluations and studies of lava tubes, only a few lava tube LiDAR datasets are currently available online. Of the nine LiDAR surveys identified by Romio et al. (2026), only five are open-access datasets, including those produced as part of the present research, while one focuses exclusively on the lava tube floor and skylight (Domínguez García-Escudero et al., 2025). This work contributes by providing a novel open-access LiDAR dataset of the Grotta di Monte Intraleo lava tube. The Unitree Go2 Edu quadruped robot platform was selected for this study as a practical and available solution with demonstrated capability for navigation in complex and uneven terrain. While platforms such as Boston Dynamics Spot or Unitree B2 would be more suitable for this type of scenario in terms of robustness and sensing capacity, they were not available for this work. To this end, a dedicated control and mapping framework was developed to enable the Go2 platform to operate in unstructured environments such as lava tubes, supporting both navigation and environmental reconstruction tasks.

The scope of this paper is limited to the integration of established technologies and methods, including LiDAR, Simultaneous Localization and Mapping (SLAM), ROS2, and photogrammetry, into a unified robotic workflow. The primary contribution is the resulting dataset from the field validation of this workflow through robotic exploration in a natural lava tube, as a planetary analog environment. Beyond its application to extraterrestrial habitat research, the dataset provides a valuable resource for the development and evaluation of robotic navigation, mapping, and localization systems for Martian and Lunar lava tubes. The data may also support research in analogous terrestrial environments, such as underground mines, hazardous caves, and tunnel networks, where similar operational challenges are encountered. Additionally, it contributes to mapping and cataloging terrestrial lava tubes (Romio et al., 2025b; Arizona State University, 2025).

The datasets generated during the presented research have been made open access and are available on https://doi.org/10.4121/778253cc-3193-4f66-bc13-03b80380424e.

## 3 Methods

**FIGURE 2:**
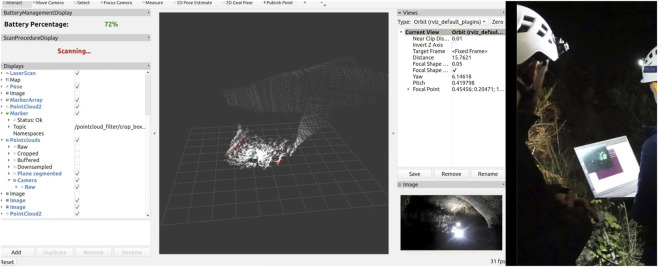
Interface of framework and local LiDAR data collection (left) and on-site deployment of the framework (right).

Based on the mission requirements related to navigation in inaccessible, unstructured and unpredictable environments, a control framework for the Unitree Go2 robot has been designed (Figure 2) to achieve the goal of scanning and measuring the environment. A new path-planning algorithm was developed to create an efficient path to scan and measure the area (Becoy et al., 2025). First, a 2D map is created by SLAM for navigational purposes, which is informed by LiDAR, which measures the actual environment in real time. This map is then used to create a graph of points-of-interest (POIs) and transform it into a topological skeleton based on its geometry.

The purpose of SLAM was to enable robot navigation. Since the robot essentially moves on a surface (even though not a plane) and since the height differences were relatively small, a 2D representation was sufficient for this purpose. However, the measurement/mapping of the cave geometry was done in 3D. The high-level path planner is informed by the graph, which creates an efficient path of POIs referred to as waypoints to be visited by the robot. The robot traverses through the waypoints using Nav2 as a local path planner, which allows avoiding nearby obstacles. A Finite State Machine (FSM) was to control the high-level operation and switch between actions, for instance, to check whether each waypoint is complete, to traverse to the next waypoint in the path, and to detect human operator input. The communication between the modules was carried out via ROS 2 using an extended interface to Unitree Go2. Additional sensors were mounted on the robot to measure other ambient parameters (such as temperature and humidity), and a Computer Vision (CV) data collection approach has been implemented, using a camera mounted on the robot.

### 3.1 Robot hardware and ROS 2 interface

The Unitree Robotics Go2 Edu quadrupedal robot has three degrees of freedom in each of its four legs and a battery life of 2–4 h with fast charging capability. However, the distance covered can be highly variable depending on the difficulty of the terrain and the amount/types of movements the robot performs. It has an inertial measurement unit (IMU) and foot-end force sensor as its proprioceptive sensing, and a HD wide-angle camera and a 4D (3D position and 1D greyscale) laser time-of-flight (TOF)-based LiDAR (Unitree L1) as its exteroceptive sensing. The LiDAR has a field-of-view (FOV) of 360° and 90°, a measurement accuracy of ± 2.0 cm, a scanning distance of 30 m with 90% reflectivity, a nearby blind area of 0.05 m, and a sampling frequency of 43,200 points/s, and a scanning frequency of 11 Hz. In addition, it is equipped with an expansion dock including an NVIDIA Jetson Orin and a manual two-handed joystick controller and supports ROS 2 communication. The ROS (Robot Operating System) is an open-source framework that provides tools, libraries, and conventions for robot simulation, hardware abstraction, and inter-process communication, enabling complex robotic applications. This significantly accelerates the prototyping and development process.

To accommodate autonomous navigation and terrain mapping, a ROS 2 framework has been developed. The point clouds of the surroundings that are measured by LiDAR are used to create a 2D navigation map of the environment using Slam Toolbox, which is a ROS library that provides SLAM of the environment in 2D. In addition, the Nav2 framework is used to enable local planning and velocity-based navigation using the 2D navigation map, given the robot’s current position and destination. To visualize data and robot states with respect to the 2D navigation map for the operator, ROS 3D Robot Visualizer (RViz) has been used, which can also visualize 3D point clouds, camera feeds in 2D images, 2D and 3D markers, and other types of visible data.

### 3.2 Terrain mapping and ambient sensing

The LiDAR is pointed toward the ground, hence, most of the points in a sampled point cloud reconstruct the terrain ahead and obstacles near the ground. This is corrected by having the robot sit down, allowing the LiDAR to point higher and create a 3D reconstruction of the environment ahead. The robot has been mapping the lava tube while stationary and subsequently moving from one scanning location to another. In addition, due to occlusions caused by the asperity, the robot had to rotate around its own axis at the scanning positions so the environment could be mapped from several angles. The LiDAR samples about 3,927 points per measurement. This implies that the robot must remain stationary until a sufficient number of points have been collected to achieve a comprehensive reconstruction of the terrain. Therefore, to achieve an optimal 3D reconstruction of the lava tube, the robot systematically maps the terrain as a 3D point cloud by alternating between standing up and sitting down to capture data for several orientations at every reachable scanning location. The number of orientations is set as a parameter that discretizes a full 360° rotation. Furthermore, images of the view from the front-facing integrated camera are captured at each scanning location. The collected images form the potential input data for future work on photogrammetry models. The robot aims to collect 360-degree data around each waypoint, with sufficient overlap between the images, so they are suitable for photogrammetry processing algorithms. The 3D point clouds are stored in. pcd file format, and the 2D images in. jpeg format.

Additionally, the background noise that can negatively affect the sensors’ performance has to be considered. For instance, humidity in lava tunnels is typically higher than outside (She et al., 2025), which increases the noise due to scattering and absorption from the water droplets in the air. To account for this, the background noise is measured in order to decrease the uncertainty in the terrain mapping. Ambient sensors are added onto the robot to measure the environment’s temperature and humidity, and the light intensity for 2D images.

### 3.3 Robot path-planning and decision-making

In order to achieve optimal 3D-reconstruction within the constraints of the weeklong mission, battery limitations, and the LiDAR’s range of 30 m, the path-planning algorithm by Becoy et al. (2025) was developed as the topological skeleton of the geometry of the free space in the 2D navigation map, which was first tested in a lab environment. This facilitated an efficient path consisting of waypoints spread evenly across the space for the robot to scan the entire space from a variety of positions. At each waypoint, the robot maps the surroundings from several orientations. This includes LiDAR scans from different orientations and data collection for photogrammetry models in the form of images taken by the integrated camera on the robot. The distance between two waypoints is set as a parameter, where the minimal distance is based on the 2D navigation map’s resolution. A decision-making policy is essential for managing the robot’s transitions between two operational modes: mapping and navigation. An FSM is employed to control the robot’s movement towards the next waypoint to achieve this. Upon receiving confirmation that the robot has reached the desired location within a specified tolerance for position and orientation, the FSM activates the terrain mapping procedure. In cases where the robot encounters difficulties traversing challenging terrain, the FSM is switched to a manual control mode when it detects a velocity input command from the human operator, allowing a human operator to temporarily take over navigation until the robot reaches the intended target location. Once all waypoints have been visited, the FSM directs the robot to return to its starting position.

### 3.4 Backup data

**FIGURE 3:**
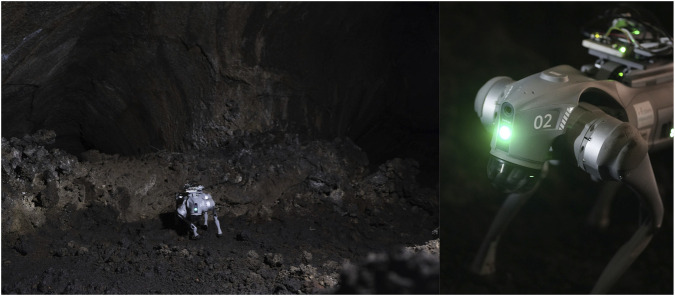
The legged Unitree Go2 Edu robot deployed inside the lava tube, mapping the environment using LiDAR (left). A detailed view of the robot (right) highlights the LiDAR sensor mounted beneath the head, the green status indicator light, and the integrated camera positioned above the light.

In addition to the abovementioned measurements, supplementary manual scans were acquired on-site as a backup in case any issues arose during the robotic mapping. Several videos of the lava tube interior and floor were recorded using iPhone 13 Pro and Samsung Galaxy A13 cameras, and additional data were collected using the Scaniverse application (Figure 3), which allows users to capture and process 3D data directly on mobile devices by employing LiDAR, photogrammetry, or Gaussian splatting techniques. As described further in Section 5 of the paper, due to issues with the onboard camera of the quadruped, these additional backup data provided material that enabled the successful testing of a photogrammetric mapping approach, which would otherwise not have been possible because of unforeseen issues that arose during the mapping operations.

## 4 Mapping results and dataset

### 4.1 LiDAR scanning

For the LiDAR data collection, the scanning position resolution is influenced by the range of the sensors. The minimal scanning position resolution is determined by capturing at least one point per half of the LiDAR range in areas with relatively flat surfaces. For surfaces that are more textured or three-dimensional, additional scanning positions are required to ensure comprehensive coverage. During the preparation and off-site testing phase of the project, it was initially assumed that all sections of the lava tube floor would be accessible to the robot. However, during the on-site testing, it became evident that the robot could only navigate a narrow path from the beginning to the end of the lava tube where obstacles were smaller than 50 mm. This limitation rendered semi-automatic waypoint programming unrealistic.

**FIGURE 4:**
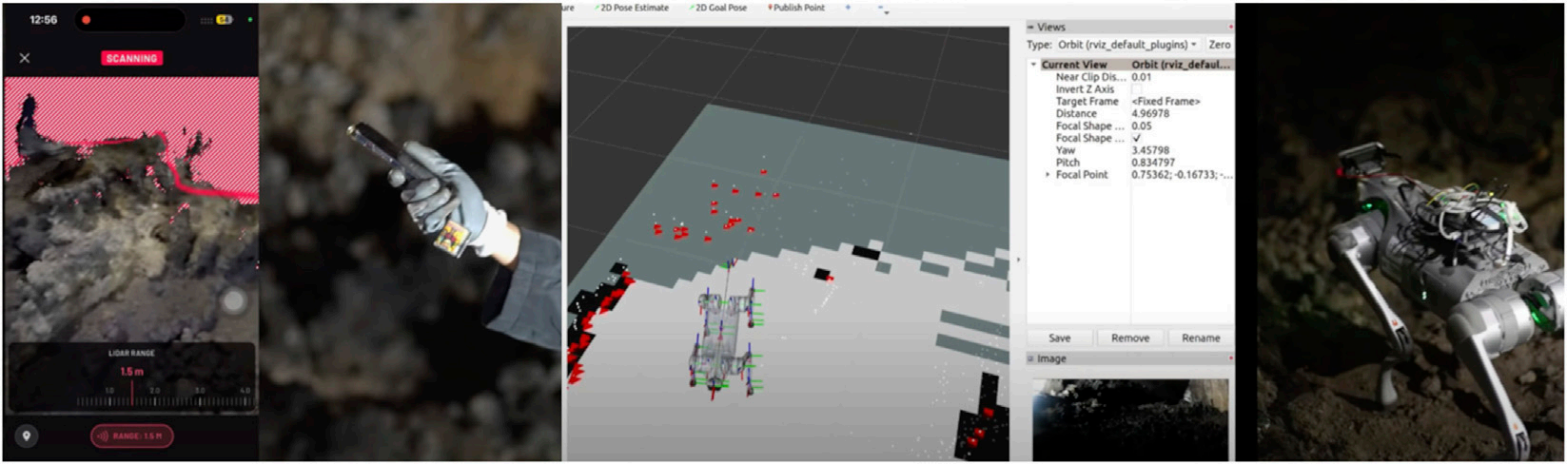
Data collection with Scaniverse (left), LiDAR data collection interface (middle), and Robot collecting LiDAR data in scanning position (right).

**FIGURE 5:**
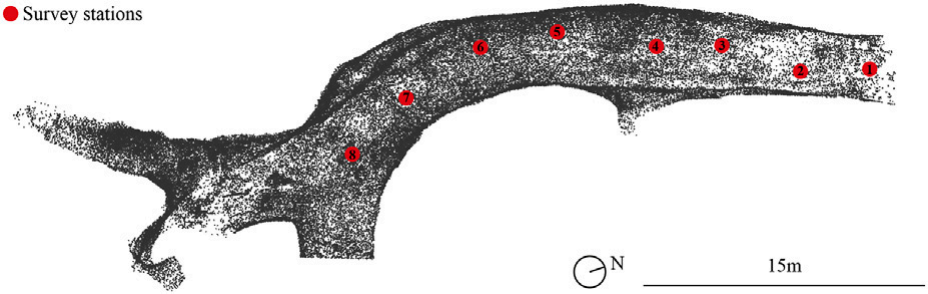
Complete LiDAR map of the lava tube with registered LiDAR point cloud, with scanning positions overlayed.

While testing autonomous navigation, it was observed that the robot’s navigation system was overly sensitive to small obstacles and sloping terrain, preventing smooth movement. However, the robot could navigate this challenging terrain (Figure 4) when manually controlled. Terrain features constrained robot navigation to a narrow path along the longitudinal axis of the lava tube. Nevertheless, LiDAR-based mapping remained effective along this trajectory, as the sensor range was sufficient to capture the full width of the lava tube. As a result, the mission strategy was adapted to rely on manual navigation to reach carefully selected scanning positions on the path that fell within the navigation ability of the robot. The waypoint selection was guided by several practical constraints. Waypoints and scanning positions were chosen along this path on relatively flat areas, spaced approximately 1–3 m apart, depending on local terrain features. Figure 5 illustrates the robot’s scanning positions, which are identifiable by localized increases in point cloud density at each acquisition location.

Limited battery life necessitated a trade-off between spatial resolution and scanning duration, leading to a reduced number of scanning locations and resulting in LiDAR scans approximately every 2–4 m. Due to the sensing range of the LiDAR, this reconstruction includes areas that were not physically traversed by the robot but were captured remotely. Figure 5 shows the registered full LiDAR point cloud, with scanning positions overlayed. The complete forward exploration of the lava tube, up to the split section that the robot could not reach due to the higher lava tube levels, covered approximately 32 m, as calculated in Rhinoceros 3D.

### 4.2 Drift and point cloud registration

Combining the LiDAR scans presented significant challenges due to a drift in the robot’s reference frame that negatively impacted the accuracy of the composite scans. To test the severity of the drift, the robot walked about 32 m from the entrance of the lava tube to the section where the main channel splits into three parts, and then back again to the same starting position, while using SLAM mapping to create a navigation map. While the robot was walking, the reference frame of the robot shifted both in position and orientation, leading to a distorted navigation map. When the robot returned to the entrance point, the drift error in the navigation map was approximately 3.5 m (drift error data is available online: https://doi.org/10.4121/778253cc-3193-4f66-bc13-03b80380424e).

During the scanning procedure, while transitioning from the standing to the sitting position, the reference frame shifted more significantly and in unpredictable directions due to the robot moving on the loose rocks while sitting down and getting back up after the data collection. This meant the collected LiDAR data could not be combined accurately with this methodology. To address this drift issue, individual LiDAR scans from each scanning position were separately stored and successively loaded in the CloudCompare software, where they were individually cleaned from noise, manually aligned, and registered using the Iterative Closest Point algorithm (ICP).

**FIGURE 6:**
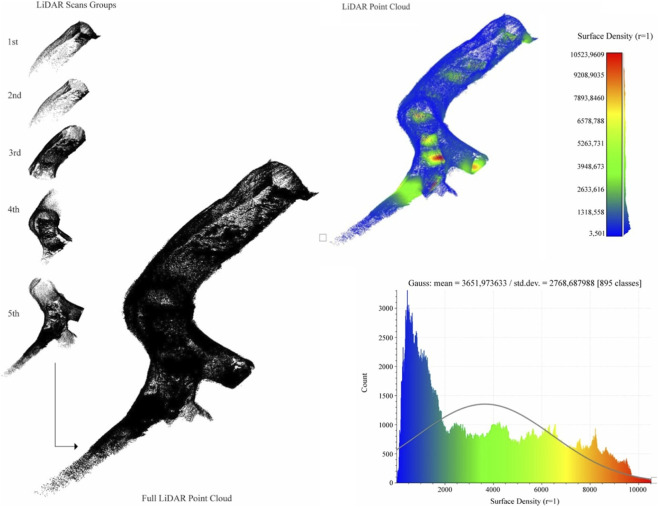
Groups of point clouds registered to obtain the final LiDAR point clouds. On the left, the five groups of sit/stand scans, registered into the final LiDAR point cloud are displayed. On the right, computed LiDAR point cloud densities and derived statistical parameters.

After the on-site acquisition, the LiDAR scans in .pdc format were imported and processed into the CloudCompare environment. First, all sit-stand pairs were identified, segmented, cleaned from noise using CloudCompare’s noise-removal tools, and registered with each other. The resulting scans were then divided into five groups, each corresponding to a different portion of the lava tube (Figure 6). The LiDAR scan groups were subsequently registered using CloudCompare’s ICP fine registration tool. A theoretical overlap of 20% was set for all group pairs, except for the registration between Groups 4 and 5, for which the overlap was reduced to 10%. To monitor the ICP registration error, the final Root Mean Square Error (RMSE) for each pairwise registration, namely between each scan group and the subsequent one, was extracted from CloudCompare’s Console. In addition, to further assess the quality of the registration, a Cloud-to-Cloud distance analysis was performed for each registered pair of point cloud groups. The resulting values are reported in Table 1.

**TABLE 1:** This table show the results of the ICP error analysis reporting both the RMS values retrieved from the logs of CloudCompare’s ICP fine alignment function and by performing a cloud-to-cloud distance analysis, for each pair of registered LiDAR point clouds, utilizing CloudCompare’s native function. Statistical parameters were then computed on the resulting cloud-to-cloud distance scalar fields.

Point cloud section/Group	ICP RMS (m)	Mean error (m)	Standard deviation (m)
1–2	0.036	0.065	0.067
2–3	0.066	0.276	0.276
3–4	0.091	1.673	1.606
4–5	0.025	1.194	0.815

Eventually, additional information regarding point cloud density was obtained by computing the surface density using CloudCompare’s Compute Geometric Features command. Surface density expresses the local number of points per unit area. For each point, a spherical neighborhood with a radius of 1 m was considered, and the number of neighboring points within this radius was divided by the corresponding local neighborhood surface, approximated as a disk of area $\pi R^{2}$ . Since R was set to 1 m, the resulting values represent the local point density in points per square meter (Figure 6). This workflow produced the final point cloud of Grotta di Monte Intraleo, which was then used to generate a 3D mesh in CloudCompare. The mesh was later exported to software such as Rhinoceros 3D for further analysis and for use as the environmental model in the Rhizome 2.0 lava tube habitat design workflow.

Although this method introduces some error (Table 1), based on the degree of overlap between the scans, this error is expected to be significantly smaller than the drift error. This revised approach is expected to enhance the accuracy and reliability of the resulting 3D models, while mitigating the effects of drift on the mission’s mapping objectives.

**FIGURE 7:**
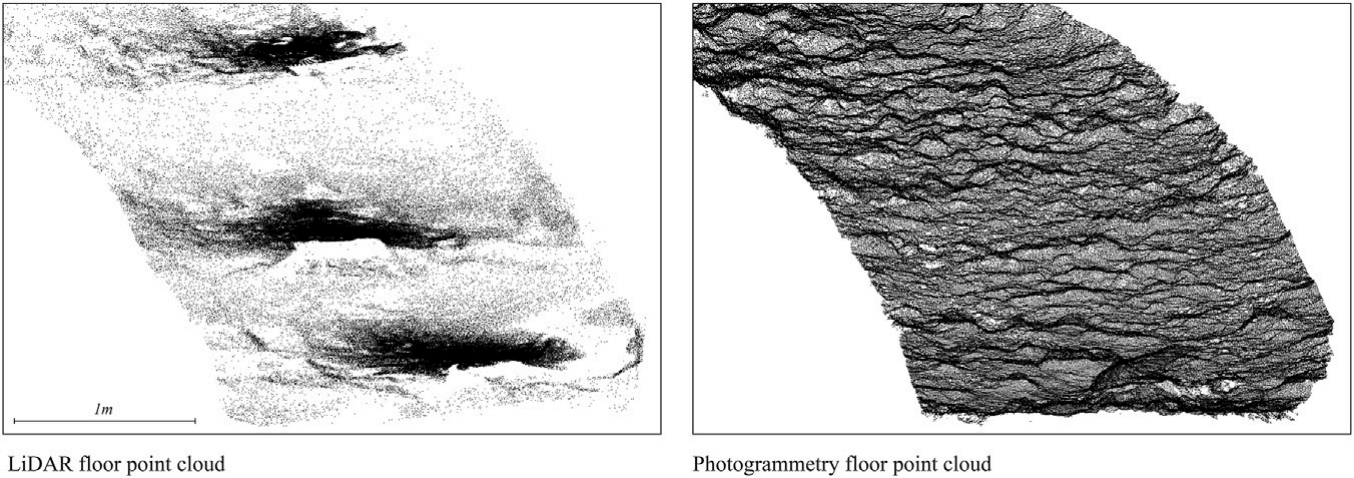
LiDAR (left) and photogrammetry data (right) of the same part of the lava tube floor. The achieved lidar point cloud with the Unitree L1 instrument is significantly less dense and fails to capture detailed floor conditions, whereas, under the mission-specific acquisition settings, photogrammetry provides a more accurate representation of the floor geometry and surface characteristics.

**FIGURE 8:**
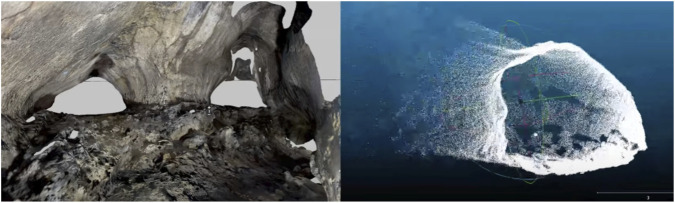
Phone scan with textures (left) and LiDAR point cloud, single scan (right).

However, during these procedures, the LiDAR floor data proved to be less dense due to an insufficient number of scanning positions for accurately capturing floor characteristics, having a total of 656,889 points. The LiDAR sensor integrated into the Unitree Go2 platform is designed primarily for real-time navigation and SLAM applications, rather than detailed geometric reconstruction. As such, a direct comparison of point density does not provide a complete assessment of sensor performance, but instead reflects differences in sensor design and intended functionality. The resulting variations are therefore best viewed as evidence of complementary sensing capabilities rather than an indication of the superiority of one modality over the other. While the scanning positions were adequate for describing the overall internal morphology of the lava tube, a denser survey would have been required to achieve a detailed mapping of the lava tube floor, which was instead more accurately represented by the photogrammetric data, in particular the one obtained with the still-frames extracted from videos recorded with the mobile phones and processed in Agisoft Metashape, which instead is composed by 1,260,001 points (Figures 7, 8).

### 4.3 Floor analysis: slope, roughness, and obstacle height

**FIGURE 9:**
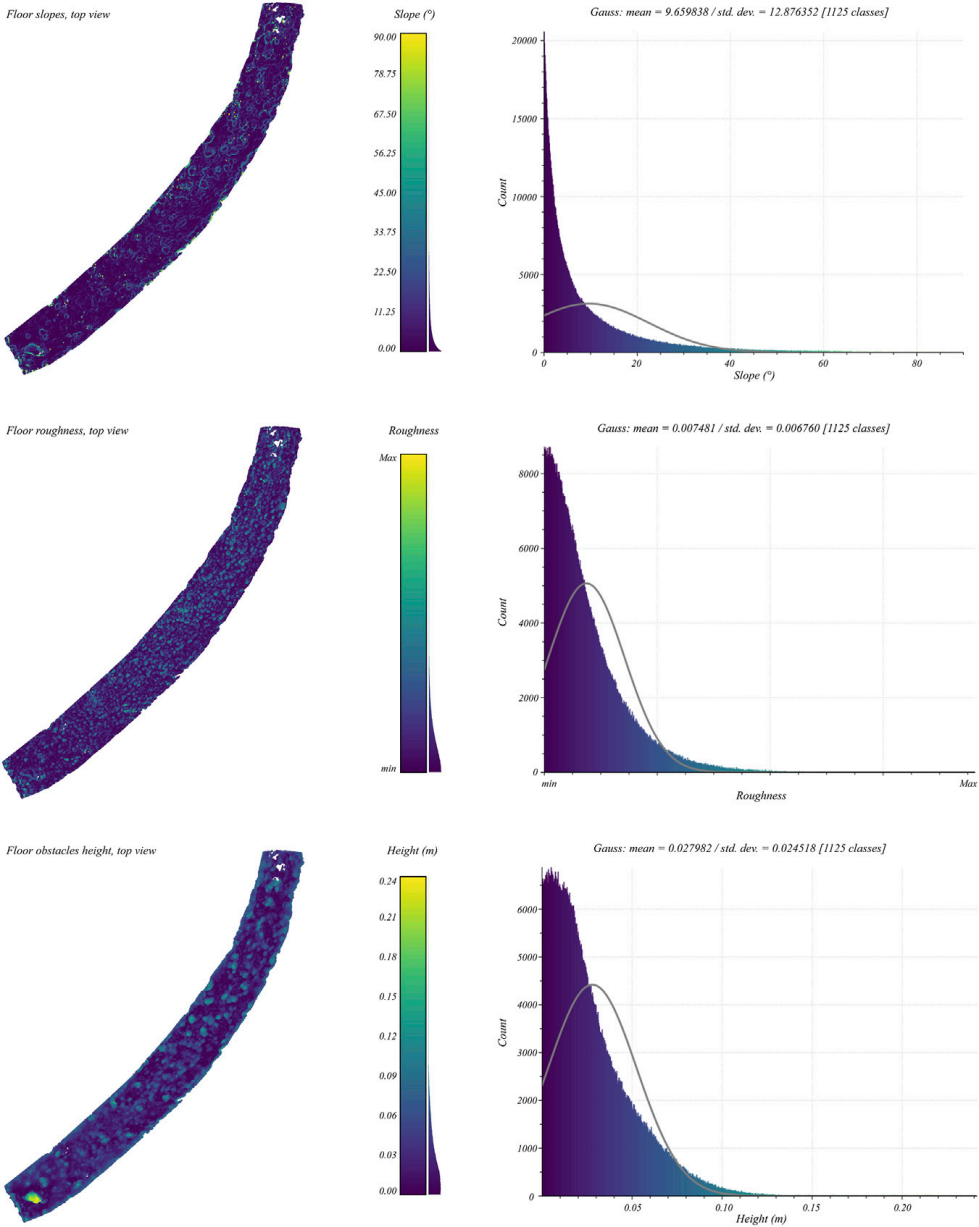
Results of the terrain characterization analyses: The maps on the left show the top view of the lava tube floor photogrammetric point cloud, colour-coded according to terrain slope, roughness, and obstacle height. On the right, the Gaussian distributions of all the analysed value classes are reported.

Figure 9 presents a detailed fragment of the point cloud corresponding to the area directly accessible to the robot and used for terrain characterization. To analyze slopes, roughness, and obstacle heights, a series of operations was performed on LiDAR and photogrammetric datasets using CloudCompare and Rhinoceros 3D. The purpose of this analysis was twofold: first, to quantitatively characterize the challenging terrain of Grotta di Monte Intraleo in relation to robotic navigation constraints; and second, to provide a preliminary assessment of floor conditions relevant to habitat suitability and future habitat planning.

As anticipated in Section 2, Grotta di Monte Intraleo represents a far-from-ideal exploration scenario, with rough and irregular floor morphologies that can constrain both robot mobility and the selection of potential settlement areas. Lava tubes with floor conditions similar to those observed in Grotta di Monte Intraleo would therefore pose significant challenges for robotic exploration, while also requiring careful terrain assessment before being considered for habitat construction. For this reason, the analysis of slope, roughness, and obstacle height helped to identify possible exploration constraints in a quantitative manner and provided a clearer overview of the floor characteristics that could inform future habitat planning.

For this analysis, the photogrammetric and LiDAR point clouds of Grotta di Monte Intraleo were first imported into CloudCompare and manually segmented to isolate the lava tube floor from the rest of the dataset. Subsequently, for each of the resulting floor point clouds, the Compute Geometric Features tool was used to derive roughness and verticality scalar fields (SFs). In CloudCompare, roughness was computed for each point as the distance between the point and the best-fitting plane calculated from its nearest neighbours. Slope was obtained by computing the verticality SF, a dimensionless value ranging from 0.0 to 1.0, which was converted into degrees by multiplying the SF by the constant value of 90 using the Scalar Fields Arithmetic tool. Once the roughness and slope scalar fields were obtained, their Gaussian distributions were computed using the Distribution Fitting tool, yielding the corresponding mean values and standard deviation.

To estimate obstacle height, the point clouds were exported from CloudCompare to Rhinoceros 3D (version 8) in .e57 file format. Once imported, the point clouds were converted into meshes using the ShrinkWrap command, and the resulting meshes were filtered to obtain a clean representation of the lava tube floor. The mesh was then smoothed to be as planar as possible, levelling local reliefs and depressions by iteratively tuning the Smooth command and applying minor manual corrections to residual bulges using the SoftTransform command. Subsequently, the QuadRemesh tool was applied to generate a mesh composed of quadrangular faces with a target edge length of 2 cm. The vertices of the resulting mesh were then extracted and exported as a. txt file to CloudCompare.

Finally, the Cloud-to-Cloud Distance tool was used to compute the absolute distances between the levelled reference point cloud and the original floor data, resulting in an obstacle-height scalar field, from which the Gaussian distribution, mean value, and standard deviation were computed using the Distribution Fitting tool.

The roughness, slope, and obstacle elevation, the results clearly indicate a challenging environment across all three parameters (Figure 9). Although the Gaussian mean values alone might suggest that the terrain is not particularly difficult for a quadruped to navigate, the relatively high standard deviation of the slope reveals a more complex scenario. Furthermore, all three output maps show that the most challenging terrain is primarily located along the sides of the lava tube, due to its morphology, and in the central part of the channel, where cooled ‘a’a-lava (see Section 2) and loose rubble of varying sizes are present. As a result, walkable areas are confined to specific longitudinal strips (Figure 9), which closely correspond to the actual path followed by the robot during the mission.

### 4.4 Lava tube scan evaluation and ground truth

A quantitative assessment of absolute mapping accuracy was not possible because no high-resolution ground-truth dataset of the lava tube exists. The only available reference is the survey map published in 1999 (Fanciulli et al., 1999), which provides a valuable representation of the cave layout but does not constitute a georeferenced three-dimensional reference model. Consequently, the quality of the reconstructed point cloud was evaluated based on the registration residuals obtained during scan alignment and through qualitative comparison with the historical survey.

The resulting reconstruction exhibits a geometry that is consistent with the documented morphology of the lava tube while providing a substantially higher level of geometric detail than the existing survey. The absence of a ground-truth three-dimensional model reflects the practical challenges associated with surveying lava tubes and similar subterranean environments. As a result, the dataset presented in this work should primarily be viewed as a new geometric reference for the surveyed section of the cave. Reconstruction quality was therefore assessed through registration error metrics and consistency with the available historical survey rather than against an absolute geometric ground truth.

## 5 Discussion

### 5.1 Mission objectives

Overall, the data collection was successful since it provides an updated dataset as an alternative to the last publicly available survey published in 1999 (Fanciulli et al., 1999). Compared to the 1999 dataset, which is limited to 2D information, our dataset includes 3D information. The main value of the dataset to the community is to facilitate research on extraterrestrial exploration.

Although localization drift limited the accuracy of the real-time SLAM reconstruction, the mission successfully achieved its primary objectives: traversing the accessible section of the lava tube, acquiring LiDAR survey data, and producing a three-dimensional reconstruction of the environment through post-processing and scan registration. The resulting dataset provides a geometric representation of the surveyed lava tube suitable for further navigation analysis and habitat assessment.

### 5.2 Drift

The drift error in the navigation map was quite significant (3.5 m based on the 32 m). The key contributor to this drift is the LiDAR sensor imperfection. The LiDAR sensor was part of the robot, which was made for scanning small areas during local obstacle avoidance and foot placement. This could be mitigated by using a better LiDAR sensor dedicated to scanning larger areas in real-time and a faster update rate when slanted terrain and foot slippage due to loose rocks are encountered. These factors induce errors in the state estimation and robot localization with respect to the environment.

### 5.3 Instability on loose rocks

One significant challenge that needs consideration is the instability of the robot on loose rocky terrain, which caused it to occasionally tip over during initial testing. Future work will explore strategies to address this issue, such as enabling the robot to first clear loose rocks autonomously. This could be achieved by equipping the Unitree robot with a robotic arm and gripper capable of performing such tasks. However, these tasks involve physical interactions with an unstructured and unpredictable environment.

To tackle this challenge, the robot manipulation system can be based on adaptive impedance control that can enable the robot to regulate how soft or stiff the arm is during the physical interaction (Abu-Dakka and Saveriano, 2020; Suomalainen et al., 2022; Peternel and Ajoudani, 2023). When approaching unknown rocks with the arm, the robotic arm could become soft to be safe in case of an unexpected collision. When a reliable and safe contact is established, the stiffness of the arm can increase to have the power to move rocks. To furnish the robot with a sufficient amount of intelligence for such behaviour, the goal is to employ generative AI, such as using models for autonomous impedance control (Jekel et al., 2026).

### 5.4 Terrain navigation

The robot’s navigation capabilities were constrained by the terrain, allowing it to move only through certain sections of the lava tube. Similar terrain characteristics, if encountered on Mars, would pose significant challenges for robotic exploration. This indicates that the developed approach and methodology could be applied to other locations with comparable geological features, providing challenging testing environments for advancing robotic lava tube mapping.

For example, Sauro et al. Bhattacharjee (2024) conducted an expedition to explore recently formed lava tubes in Iceland. The exploration of terrestrial lava tubes under extreme conditions, including very high internal air temperatures reaching up to 200 °C and toxic gases, represents a high-risk activity. In recent years, robotic exploration of such hazardous underground environments has been significantly advanced by initiatives such as the DARPA Subterranean Challenge (SubT) (Santagata et al., 2022), whose final event took place in September 2021. The competition required autonomous systems, including wheeled and legged robots as well as UAVs, to map, navigate, and search tunnel, urban, and cave environments under difficult conditions, including dust, darkness, degraded communication, and spatial complexity. These challenges closely resemble those expected in future Martian and lunar cave exploration, highlighting the relevance of terrestrial underground robotics research both for terrestrial and space applications Morrell et al. (2024); Agha et al. (2022); Tranzatto et al. (2022).

### 5.5 2D Path planning

The main limitation of the implemented path planning algorithm is that it utilizes a 2D map of the environment, whereas the actual environment has elevation changes aside from longitudinal and lateral directions. This affects the localization in SLAM, and the slanted terrain in lava tubes can be misidentified as obstacle due to projection. Moreover, it will cause issue with overlapping and multi-planar tunnels along the depth.

### 5.6 Photogrammetry data capture limitations

The data collected had several issues that warrant improvement. The onboard camera lacked a wide-angle lens, resulting in video frames with limited coverage of the lava tube. The robot recorded video while navigating from the entrance to the part of the lava tube where it split into three parts. This approach was integrated when navigating between the LiDAR scanning positions. However, the lack of video stabilization in the robot’s camera, meaning that the recorded video was blurry and noisy in combination with the rough terrain, made the data unsuitable for further processing. Hence, mobile phone data for the terrain floor analysis was used.

Another consideration is that the robot is limited to collecting data from its own point of view, which restricts its ability to capture details in areas obscured by large obstacles or regions that are inaccessible due to challenging terrain features. As a result, certain sections of the lava tunnel remain shadowed or hidden from scanning. A potential solution that will be addressed in future work to overcome this limitation is to mount the LiDAR on a robotic arm that is attached to the robot. Such an approach would extend the workspace of the LiDAR, allowing it to access and scan occluded regions. This approach could significantly increase the quantity and quality of the data collected, enhancing the comprehensiveness of the mapping process. For the data collection for photogrammetry, a camera with image stabilization could be mounted on the same arm. The robotic arm could rotate the camera, in a spiraling motion, during navigation, ensuring that the camera sufficiently captures the view of the lava tube and that enough overlap between the video data is present.

### 5.7 Rhizome 2.0: future work

The collected lava tube data will be evaluated in a follow-up paper for habitability. The suitability of a lava tube for habitation will be evaluated using a combination of the collected data for structural, environmental, and constructability metrics. These include a visual assessment of structural stability in the photogrammetry and LiDAR point cloud model (e.g., presence of large cracks or evidence of collapse), roof span, roof thickness for radiation shielding, and evidence of rockfall history. Spatial suitability is assessed through minimum tunnel width, ceiling height, emergency egress opportunities, and construction access. Robotic constructability is evaluated using reachability (fraction of the cave accessible to robotic systems) and traversability metrics, including slope, floor roughness, and floor obstacle height. Together, these metrics provide an indication of the habitability, safety, and feasibility of robotic construction within the lava tube.

Future work will focus on extending the current dataset and workflow toward more representative Martian-scale lava tube environment. A first step involves scaling and extrapolating the reconstructed geometry using a pyroduct-based simulation, enabling the creation of larger Martian-like lava tube models for analysis and testing.

On this extended geometry, systematic habitat placement evaluation will be conducted, considering constraints such as available width, usable volume, navigability of robotic platforms, and accessibility of potential infrastructure locations. In parallel, habitat design will be explored as an environment-driven process, where structural and functional requirements are derived directly from the cave geometry and optimized for robotic construction sequences and assembly order.

Additionally, a Voronoi-based componential logic will be developed, and a fragment of the habitat will be designed and prototyped using Design to Robotic Production and Assembly (D2RP&A) methodologies.

Finally, research will address the interaction between habitat structures and the lava tube floor, focusing on how component-based systems can be anchored, distributed, and structurally integrated with the irregular natural substrate.

The mapped ground surface data will also be utilized to enhance the robot’s capabilities for navigating uneven, rocky terrain, which in turn will be beneficial for improving the mapping of the terrain in the future, as well as implementing other construction tasks ranging from mining for *In-Situ* resources to prefabrication of components and their assembly.

The data resulting from this research has already proved useful in follow-up research. For example, it has been utilized for reconstructing the 3D environment of Grotta di Monte Intraleo, (Romio et al., 2025a). This data has been used, together with other open-access repositories of LiDAR lava tube datasets to validate the outputs of the Pyroduct software (Romio et al., 2025c; Romio et al., 2026), an open-access plug-in for Rhinoceros’ 3D Visual Programming Language (VPL) Grasshopper to generate realistic and parametric 3D models of Lunar, Martian and Terrestrial lava tubes. The data from this paper was used to compute the Cloud-to-Cloud distance in the Cloud Compare software between the generated lava tube models and the real LiDAR data.

## 6 Conclusion

The presented robotic mapping methodology has been successful in terms of data collection, significantly adding to available open-access lava tube datasets and specifically the Grotta di Monte Intraleo lava tube. Post-processed scan registration resulted in a coherent point cloud that captured the principal geometric features of the surveyed lava tube (Figure 4). Challenges for collecting data for autonomous mapping have been identified and will be addressed in future steps, paving the road map for future autonomous robotic terrain mapping and construction of extraterrestrial habitats.

## Data Availability

The datasets presented in this study can be found in online repositories. The names of the repository/repositories and accession number(s) can be found in the article/supplementary material.
